# Trend of lipid and thyroid function tests in adults without overt thyroid diseases: A cohort from Tehran thyroid study

**DOI:** 10.1371/journal.pone.0216389

**Published:** 2019-05-16

**Authors:** Salma Ahi, Atieh Amouzegar, Safoora Gharibzadeh, Hossein Delshad, Maryam Tohidi, Fereidoun Azizi

**Affiliations:** 1 Endocrine Research Center, Research Institute for Endocrine Sciences, Shahid Beheshti University of Medical Sciences, Tehran, Iran; 2 Department of Epidemiology and Biostatistics, Research Center for Emerging and Reemerging Infectious Diseases, Pasture Institute of Iran, Tehran, Iran; 3 Metabolic Research Center, Research Institute for Endocrine Sciences, Shahid Beheshti University of Medical Sciences, Tehran, Iran; Boston University School of Medicine, UNITED STATES

## Abstract

**Context:**

While the role of overt hypothyroidism in lipid disorders is clear, the association between dyslipidemia and subclinical thyroid diseases remains unclarified.

**Objective:**

To examine lipid trends based on thyroid function over a 10-year period.

**Design:**

This is a prospective population based cohort study.

**Setting:**

General community.

**Participants:**

2383 euthyroid participants, as well as those with subclinical thyroid diseases, in all residents of district 13 of Tehran were examined. Subjects who were on levothyroxine, anti-hyperthyroid drugs, and glucocorticoids, those with a history of thyroid surgery or RAI and pregnant women were excluded.

**Main outcome measures:**

Lipid trends in Model 1 were adjusted for age and follow up duration, and in Model 2 gender-specific multivariate adjustments were performed for thyroid status, diabetes mellitus, smoking status, education, BMI, lipid lowering medications, age and follow up duration by using generalized estimating equations.

**Results:**

In every four years of assessments, there were significant decreases in levels of all lipid parameters (all Ps <0.001) except for HDL-C, in which a decrescendo-crescendo trend was observed. The results did not change after adjusting for thyroid status, consumption of lipid lowering drugs during the follow-up period, or other variables. There were significant decreases in the prevalence of hypercholesterolemia and hypertriglyceridemia (all Ps <0.001) during the follow-up period.

**Conclusion:**

During a 10 year follow-up, decrescendo trends were observed in levels of total cholesterol, triglycerides, which were not be accounted for by the consumption of lipid lowering drugs and thyroid status.

## Introduction

Dyslipidemia is the most easily modifiable risk factor for cardiovascular disease; early detection and treatment of lipid disorders hence, may reduce the risk of cardiac events or delay them [1]. Because of the high prevalence of cardiovascular diseases and the economic burden they impose, research on identifying risk factors for such diseases is of utmost importance [2]. Both hyperlipidemia and subclinical thyroid disorders are common. Prevalence of subclinical hypothyroidism is 2.1–4.1% in different populations and is two to three fold higher in patients with hyperlipidemia [3, 4]. The American thyroid association recommends thyroid function tests in all patients above 35 years old [5]. Similarly, thyroid function should be checked in all patients with dyslipidemia [6]. While the role of clinical hypothyroidism in lipid disorders is clear, the association between dyslipidemia and subclinical thyroid diseases remains unclarified [7] several studies showed significant changes in lipid parameters of participants with subclinical hypothyroidism [8–12] but the relationship between TSH in the normal range with serum lipid changes weren’t observed in other studies [13–17]. Previous studies in this regard almost checked TSH at baseline with various definition of normal ranges in the cross sectional design [8, 10–17]. Various ranges of TSH that were used for the definition of Euthyroidism and subclinical thyroid disorders in different studies leads to miscellaneous results [8–17]. The necessity of comprehensive cohort for evaluation of lipid trends in Euthyroids and subclinical thyroid disorders is clear due to literature controversies and limitations, The purpose of our study was to evaluate lipid trend and its correlation with thyroid function in the Iranian population.

### Study population

The present study was conducted within the framework of the Tehran Thyroid Study (TTS), a prospective population-based cohort study performed on residents of district-13 of Tehran with the aim of evaluating the prevalence and natural course of thyroid diseases and their long term consequences in terms of metabolic and ischemic heart disease, cardiovascular and all-cause mortality in the urban, iodine sufficient population of Tehran [18]. The TTS is a subgroup study of the Tehran Lipid and Glucose Study (TLGS), which is a prospective population-based cohort study of over 15,000 residents from district 13 of Tehran, being conducted with the aim of determining the prevalence and incidence of risk factors for non-communicable diseases [18]. For the TLGS initially, a total of 15,005 individuals, aged≥ 3 years, under coverage of 3 medical health centers in Tehran, were selected by multistage stratified cluster sampling; from those 10,368 participants, aged ≥20 years; 5783 were selected to participate in the Tehran Thyroid Study. Details of the study design assessments and data collection have previously been published elsewhere [18].

At the first visit, the study was explained to subjects and demographic data were obtained. All clinical examinations were performed at the beginning of the study and collection of demographic, clinical and laboratory data was repeated every 3 years [18], [19].

The study protocol was approved by the Human Research Committee of Shahid Beheshti University of Medical Sciences. Written consents were obtained at the beginning. This study was conducted in four phases [12, 13].This study initiated in 1999(1st phase from 1999–2001) and followed for 12 years (2^nd^ phase: 2002_2005), (3^rd^ phase: 2005_2008), (4^th^ phase: 2008_2011).

**Fig 1:**
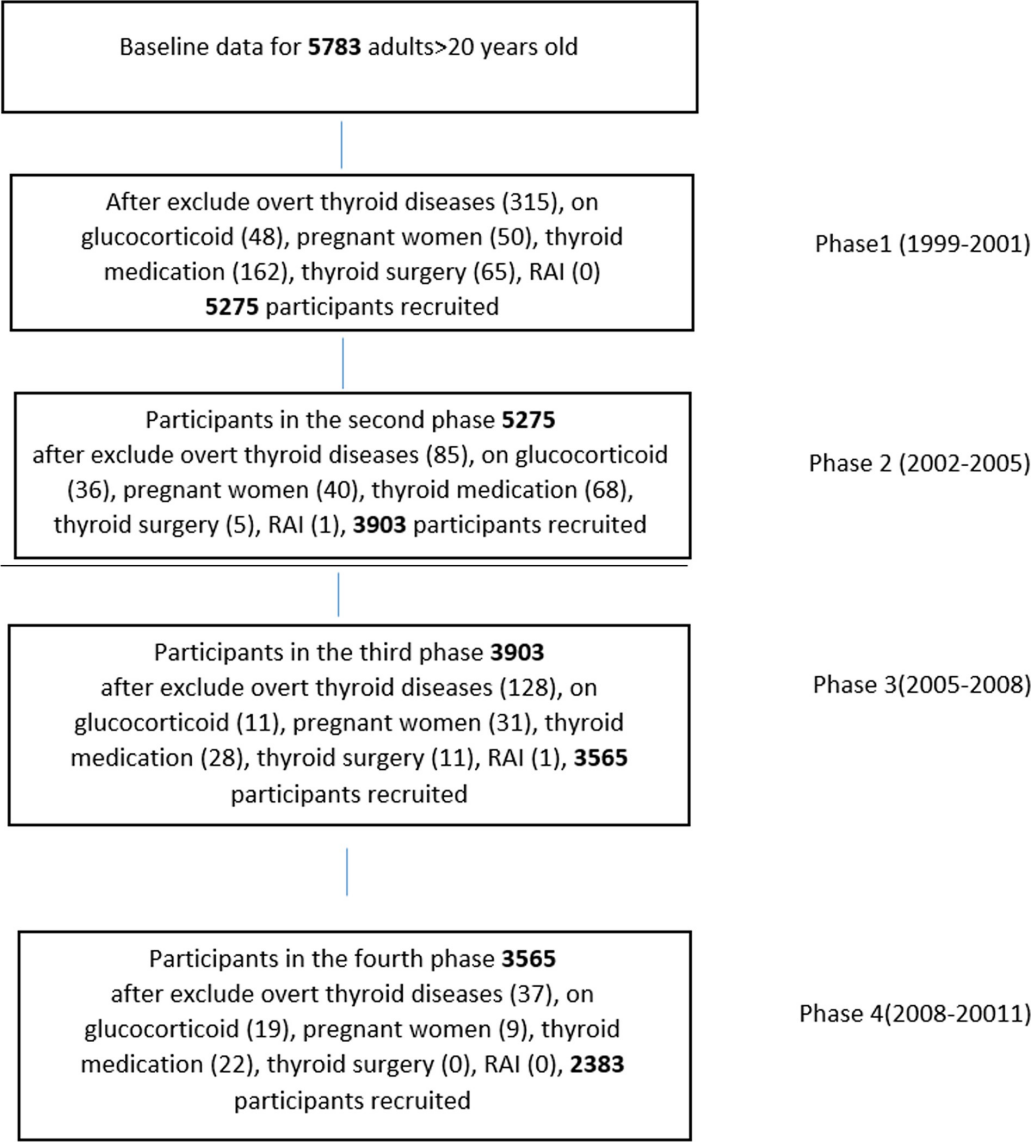
Study population.

5783 Euthyroid individuals and those with subclinical thyroid diseases were recruited at baseline and at each subsequent phase. Subjects who were on levothyroxine, anti-hyperthyroid drugs, and glucocorticoids, those with a history of thyroid surgery or RAI and pregnant women were excluded from the study at each phase. Eventually 2,383 participants were enrolled and studied in four phases (Fig 1).

### Clinical and anthropometric measurements

Data regarding age, sex, past medical history of cardiovascular disease, medications, and smoking status were collected using pretested questionnaires. Anthropometric measurements, including weight and waist circumference (WC) were collected according to standard protocols. Waist circumference was measured at the level of umbilicus by a trained technician. Body mass index (BMI) was calculated as weight in kilograms divided by height in meters squared. Blood pressure (BP) was measured twice in the sitting position, after resting for 15 minutes, using a standard mercury sphygmomanometer. Education status was divided into three categories: Illiterate or primary school (less than 6 years); high school (6–12 years) and higher than high school (over 12 years). Marital status was categorized: single, married and widowed/divorced.

### Laboratory measurements

Venous blood samples were collected from the study participants between 07:00 and 09:00 AM after 12–14 hour overnight fasting and samples were centrifuged within 30–45 min of collection. Fasting plasma glucose (FPG) was measured by the enzymatic colorimetric glucose oxidase method. Inter-and intra-assay coefficients of variation (CV) at baseline and follow-up phases were both less than 2.3%. A Selectra 2 autoanalyzer (Vital Scientific, Spankeren, Netherlands) was used to analyze samples for serum total cholesterol (TC) and triglycerides. Enzymatic calorimetric tests were used to assay TC with cholesterol esterase and cholesterol oxidase and glycerol phosphate oxidase was used for triglycerides. HDL-C was measured after precipitation of the lipoprotein-B-containing lipoproteins with phosphotungstic acid. LDL-C was calculated with the modified Friedewald formula [20]. Non-HDL-C was calculated by subtracting HDL-C from TC; TC/HDL-C and TG/HDL-C were calculated by dividing TC and TG by HDL-C, respectively. Both inter and intra-assay coefficients of variation were less than 1.9%, 2.1%, and 3% for TC, TGs and HDL-C, respectively, in baseline and follow-up assays of lipid profile. The study reference ranges for thyroid function tests were the same as those used for Tehran Thyroid Study (TTS) data [18, 19]. TSH and free thyroxin (FT4) were measured on -70ºC stored serum samples by the electrochemiluminescence immunoassay (ECLIA) method, using Roche Diagnostics kits and Roche/Hitachi Cobas e- 411 analyzer (GmbH, Mannheim, Germany). To monitor the accuracy of assays lyophilized quality control material (Lyphochek Immunoassay plus Control, Bio-Rad Laboratories) was used. Intra- and inter-assay CVs were 1.3% and 3.7% for FT4 and 1.5% and 4.5% for TSH determinations, respectively. Immunoenzymometric assay (IEMA) by the related kit (Monobind, Costa Mesa, CA, USA) and the Sunrise ELISA reader (Tecan Co., Salzburg, Austria) were used for thyroid peroxidase antibody (TPO-Ab). TPO-Ab intra- and inter-assay CVs were 3.9% and 4.7%, respectively [12]; TPOAb > 40 IU/mL was considered positive, based on used kits [19].

### Definition

Clinical hypothyroidism was defined as being on levothyroxine treatment and having TSH >5.06 mU/L and FT4< 0.91. Clinical hyperthyroidism was defined as being on Anti-thyroid medications or having a TSH<0.32mU/L and FT4>1.55 ng/dl. Subclinical hypothyroidism was defined as TSH>5.06mU/L and normal FT4 between 0.91and1.55 ng/dl. Subclinical hyperthyroidism was defined as TSH<0.32mU/L and FT4<1.55 ng/dl [19].

Hypercholesterolemia was defined as serum TC ≥ 240mg/dl, hypertriglyceridemia as serum TGs ≥ 200mg/dl and Low HDL-C as serum HDL< 40mg/dl. High non-HDL-C was defined as serum non-HDL-C ≥ 190mg/dl. TC/HDL-C ratio ≥ 5.97 and TG/HDL-C ratio ≥ 2.18 were considered elevated [15]. Hypertension was defined as a systolic blood pressure ≥ 140mmHg or a diastolic blood pressure ≥ 90 mmHg. Patients on antihypertensive agents were also considered to be hypertensive. Diabetes mellitus was defined as FPG ≥ 126 mg/dl or the patient being on a glucose lowering medication. Participants were considered current smokers if they smoked cigarettes at the time of enrollment or at each predefined stage.

### Statistical analysis

Due to the limited number of participants with subclinical thyroid diseases, it was impossible to study lipid trends in each category of patients with subclinical thyroid disease. Therefore lipid trends and the trend of dyslipidemia were adjusted based on participants’ thyroid function tests. Means and SD were used to describe normally distributed variables. Medians and inter-quartile ranges were used to describe TG levels and TSH because the distributions were not normal. All analyses were performed separately for men and women.

Standard T-test was used to compare the differences in means of normally distributed variables and Chi-squared test for categorical variables. The Kruskal-Wallis test was used for non-normally distributed variables such as TGs and TSH.

Baseline characteristics including age, education, marital status, smoking status, systolic blood pressure, WC, diabetes, and the outcomes were included in the logistic regression model. After eliminating the participants who were lost to follow up in each phase, lost data were limited as shown in the following S1 Table. Because of the small number of patients lost to follow up, estimation of selection bias and developing propensity scores was not necessary and selection bias hence is unlikely to have affected our estimations.

Trends of TC, LDL-C, HDL-C, and TGs levels and the trend of non-HDL-C, TC/HDL-C, TG/HDL-C ratios were examined. Trends of lipid parameters of the population over time were examined using generalized estimating equations (GEE), a method, which facilitates analysis of longitudinal data or repeated measures on dependent variables of many different distributions, mainly binary data [21]. It is a statistical technique that enables researchers to restrict modeling to the first moment, which requires only the correct working correlation matrix, to specify the univariate marginal distributions [22].

Two models were used: The age-adjusted (for age, propensity score, and the study phase) and the multivariate-adjusted (for age, examination cycle, propensity score, BMI, current smoking, diabetes, hypertension, thyroid function tests and total cholesterol level). Both models were performed for males and females separately.

Trends in prevalence of dyslipidemia were examined for high cholesterol, high TGs, low HDL-C, high non-HDL-C, high TC/HDL-C and high TG/HDL-C. Pooled logistic regression was used to describe the significance of trends in the proportions. All analyses were performed on individuals who participated in all 4 phase follow up visits. Analyses were repeated and adjusted separately for thyroid functions. Separate analyses of lipid trends based on thyroid function were performed in the following four groups: Q1 those with the lowest levels of TSH within the normal range (TSH < 0.91 mU/L), Q2 those with 0.91 < TSH < 1.46 mU/L, Q3 those with 1.46 < TSH < 2.25 mU/L, Q4 those with the highest level of TSH within the normal range (TSH >2.25 mU/L). P-values< 0.05 were considered significant. All analyses were performed using SATA 12 statistical software.

## Results

Mean ages of male and female study participants were 39.1 and 41.5 years, respectively; 56% of the participants were female. The mean BMI was 27 kg/m^2^ for women and 25.7 kg/m^2^ for men. Median TSH value was 1.85 mU/L in women and 1.40 mU/L in men. There were significant differences in baseline blood pressure, educational level, WC, BMI, antihyperlipid medications, smoking, lipid parameters, and thyroid function tests between men and women. All baseline characteristics except for FPG, MLDL, and diabetic population, were significantly different between men and women (Table 1).

**Table 1:** Baseline characteristics of men and women participants.

	*Men(2275)*	*Women(3000)*	*Pvalue*
Age، y*	***41*.*5±15*.*1***	***39*.*1±13*.*7***	***<0*.*001***
Systolic BP، mm Hg*	***118±16*.*8***	***115±18*.*4***	***<0*.*001***
Diastolic BPmm Hg*	***76*.*6±10*.*7***	***75*.*9±10*.*5***	***0*.*01***
***Lipid lowering drug treatmet،*** ‡	***48(2*.*11)***	***105(3*.*50)***	***<0*.*001***
Fasting glucose، mg/dL*	***96*.*5±26*.*9***	***95*.*5±31*.*1***	***0*.*24***
***Diabetics*** ‡	***216(10*.*1)***	***289(10*.*4)***	***0*.*75***
***Education*** ‡			***<0*.*001***
***Primary(<6years)***	***411(18*.*07)***	***751(25*.*03)***	
***Secondary(6-12y***	***937(41*.*19)***	***1220(40*.*67)***	
***Higher(>12years)***	***269(11*.*82)***	***204(6*.*80)***	
***BMI، kg/m***^2^*	***25*.*7±4*.*12***	***27*.*0±4*.*7***	***<0*.*001***
WC ،cm*	***89*.*2±11*.*3***	***86*.*1±12*.*5***	***<0*.*001***
***Smoking***‡	***373(16*.*39)***	***56(1*.*86)***	***<0*.*001***
Total cholesterol، mg/dL*	***197±41*.*7***	***203±47*.*2***	***<0*.*001***
LDL-C، mg/dL modified*	***126±33*.*3***	***127±37*.*7***	***0* .*12***
HDL-C، mg/dL*	***37*.*6±9*.*09***	***44*.*5±1*.*0***	***<0*.*001***
***TG، mg/dL†***	***149 (102–214)***	***122(84–184)***	***<0*.*001***
***TG/HDL-C†***	***4*.*12 (2*.*5–6*.*4)***	***2*.*85(1*.*78–4*.*56)***	***<0*.*001***
***TC/HDL-C†***	***5*.*30(4*.*33–6*.*45)***	***4*.*57(3*.*68–5*.*62)***	***<0*.*001***
***TSH(IU/ml)*** ***†***	***1*.*4 (0*.*89–2*.*13)***	***1*.*85(1*.*11–2*.*97)***	***<0*.*001***
***Tpo Ab (>40IU/ml)*** ‡	***173(7*.*6%)***	***422(14*.*1%)***	***<0*.*001***
FT4(ng/dl)*	***1*.*27±0*.*18***	***1*.*17±0*.*17***	***<0*.*001***

Abbreviations: BMI body mass index, BP blood pressure, FPG Fasting plasma glucose, TC total cholesterol, HDL-C high-density lipoprotein cholesterol, non HDL-C non-high-density lipoprotein cholesterol, TG triglycerides, WC waist circumference Values are presented as mean (SD) unless otherwise indicated

‡ n (%)

† Median (IQ)

*p<0.05 is significant

Baseline characteristics of male and female participants were recorded separately. Male participants were older, more likely to be hypertensive and be active smokers, had higher educational levels, as well as higher TGs, TC/HDL-C, TG/HDL-C and FT4 levels compared to the female participants. In comparison, female participants had a higher mean BMI, total cholesterol, HDL-C, TSH, and positive TPOAb percent (Table 1). The rate of change in serum lipid levels and thyroid function tests are reported in Table 2. In male participants the most changes in total cholesterol, MLDL, and TG were between the first and the second phases of and the most significant changes in TSH and TPOAb were observed between the third and the fourth phases of the study. In female participants the most significant changes in TC, MLDL and FT4 levels occurred between the first and the second phases of the study, while the biggest changes in HDL-C and FT4 levels occurred between the third and the fourth phases of the study (Table 2). Maximum variations in TC, MLDL, and HDL in female participants were higher than those in male participants (Table 2).

**Table 2:** Rate of change in serum lipid levels and thyroid function tests (mg/dl/phase).

	*Phase I-II* *baseline vs 3ys*	*Phase II-III* *3ys vs 6ys*	*Phase III-IV* *6vs9ys*
***Men***			
***Total cholesterol(mg/dl)***	***-9*.*5±27*.*9***	***0*.*96±27*.*60***	***0*.*25±31*.*28***
***Triglycerides(mg/dl)***	***-4(-42،32)***	***-1*.*0(-34،32)***	***-3*.*00(-40،29)***
***HDL-cholesterol(mg/dl)***	***-1*.*66±8*.*11***	***3*.*18±7*.*03***	***5*.*02±6*.*77***
***M*.*LDL-cholesterol(mg/dl)***	***-6*.*41±22*.*54***	***-1*.*74±22*.*50***	***-3*.*51±24*.*84***
***TSH(IU/ml)***	***0*.*10(0*. *22،0*.*47)***	***0*.*14(-0*.*21،0*.*55)***	***0*.*24(-*.*13،0*.*60)***
***FT4(ng/dl)***	***-0*.*01±0*.*12***	***-0*.*01±0*.*11***	***-0*.*01±0*.*10***
***Tpo Ab(>40IU/ml)***	***-0*.*09(-0*.*86–0*.*63)***	***0*.*00(-0*.*67،0*.*73)***	***0*.*18(-0*.*33،0*.*99)***
***Women***			
***Total cholesterol(mg/dl)***	***-11*.*42±31*.*21***	***0*.*70±31*.*90***	***0*.*89±34*.*55***
***Triglycerides(mg/dl)***	***-2(-33،30)***	***-1*.*00(-31،28)***	***-4(-35،25)***
***HDL-cholesterol(mg/dl)***	***-2*.*37±10*.*19***	***4*.*02±9*.*17***	***6*.*65±8*.*30***
***LDL-cholesterol(mg/dl)***	***-7*.*85±25*.*52***	***-2*.*84±25*.*4***	***-2±28*.*5***
***TSH(IU/ml)***	***0*.*12(-0*.*37،0*.*70)***	***0*.*18(-0*.*34،0*.*71)***	***0*.*24(-0*.*25،0*.*82)***
***FT4(ng/dl)***	***-0*.*01± 0*.*14***	***-0*.*01±0*.*12***	***-0*.*00±0*.*11***
***TpoAb(>40IU/ml)***	***-0*.*16(-1*.*20،0*.*73)***	***0*.*00(-0*.*86،1*.*00)***	***0*.*07(-0*.*76،1*.*05)***

A decrescendo trend of four phasic lipid parameters was observed in men and women (Table 3). As presented in Table 4 a significant decrease in prevalence of dyslipidemia was observed; hypercholesterolemia, high LDL-C, high non HDL-C, hypertriglyceridemia, high TG/HDL, and high TC/HDL decreased over time from the first to the fourth phase of the study in both sexes. A crescendo-decrescendo trend in low HDL was observed. The prevalence of hypercholesterolemia decreased by 36% in male, and by 25% in female participants. Similarly, the prevalence of hypertriglyceridemia decreased by 13% in men and by 5% in women; prevalence of low HDL also decreased by 28% in male and by 55% in female participants. We observed a 17mg/dl decrease in total cholesterol and MLDL between the 1^st^ and 4^th^ phase of the study (Table 4). Separate analyses of lipid trends based on thyroid functions were performed between TSH categories.

**Table 3:** Four phasic lipid trend.

	Men						Women					
	(baseline)Phase I	Phase II(3y)	Phase III(6y)	Phase IV(9y)	P value		Phase I(baseline	Phase II(3y)		Phase III(6y)	Phase IV(9)	P value
Age adjusted analysis b												
Totalcholesterol, mg/dL	205.31(202.92–207.70)	190.32(188.0–192.62)	188.96(186.66–191.27)	188.41(180.00–190.80)		<0.001	220.38(218.22–222.53)		200.14(198.08–202.21)	196.00(193.94–198.07)	192.62(190.47–194.77)	<0.001
HDL-C, mg/dL	38.15(37.60–38.70)	34.73(34.19–35.26)	36.98(36.45–37.51)	41.79(41.24–42.34)		<0.001	44.56(44.56–45.74)		41.05(40.49–41.61)	43.95(43.39–44.51)	50.51(49.92–51.09)	<0.001
TG, mg/dL	5.05(5.02–5.09)	5.01(4.97–5.04)	5.01(4.98–5.04)	4.98(4.94–5.01)		<0.001	4.98(4.95–5.01)		4.90(4.88–4.93)	4.87(4.85–4.90)	4.79(4.76–4.82)	<0.001
M LDL,mg/dL	132.10(130.17–134.04)	122.70(120.84–124.57)	119.44(117.57–121.30)	115.26(113.32–117.18)		<0.001	140.65(138.89–142.42)		127.34(125.65–129.03)	121.80(120.11–123.50)	114.05(112.29–115.81)	<0.001
TG/HDL-C	1.50(1.45–1.54)	1.51(1.47–1.55)	1.41(1. 37–1.45)	1.23(1.19–1.28)		<0.001	1.20(1.16–1.23)		1.22(1.19–1.25)	1.11(1.08–1.15)	0.89(0.86–0.93)	<0.001
TC/HDL-C	1.69(1.67–1.70)	1.71(1.69–1.72)	1.63(1.61–1.65)	1.50(1.49–1.52)		<0.001	1.59(1.57–1.60)		1.59(1.57–1.60)	1.50(1.48–1.51)	1.34(1.32–1.35)	<0.001
Multivariate adjusted analysis c												
Totalcholesterol, mg/dL	206.87(204.33–209.40)	190.10(187.72–192.48)	188.02(185.63–190.41)	188.08(185.36–190.80)		<0.001	217.40(214.75–220.52)		197.26(194.75–199.76)	192.56(190.05–195.08)	191.34(186.86–195.81)	<0.001
HDL-C, mg/dL	37.57(37.00–38.14)	34.45(33.91–34.98)	36.92(36.38–37.46)	42.12(41.50–42.74)		<0.001	45.37(44.62–46.12)		41.11(40.41–41.82)	44.02(43.31–44.73)	49.94(48.68–51.19)	<0.001
TG, mg/dL	5.10(5.07–5.14)	5.02(4.99–5.05)	5.00(4.96–5.03)	4.95(4.91–4.99)		<0.001	4.94(4.91–4.97)		4.87(4.84–4.90)	4.85(4.82–4.88)	4.75(4.69–4.81)	<0.001
MLDL	133.42(131.38–135.47)	122.49(120.57–124.41)	118.78(116.85–120.70)	114.88(112.69–117.07)		<0.001	138.44(136.24–140.63)		125.24(123.16–127.32)	119.06(116.98–121.15)	113.92(110.21–117.64)	<0.001
TG/HDL-C	1.20(1.16–1.23)	1.22(1.19–1.25)	1.11(1.08–1.15)	0.89(0.86–0 .93)		<0.001	1.16(1.11–1.20)		1.19(1.15–1.23)	1.09(1.05–1.13)	0.86(0.79–0.93)	<0.001
TC/HDL-C	1.71(1.69–1.73)	1.71(1.69–1.73)	1.63(1.61–1.64)	1.49(1.47–1.51)		<0.001	1.57(1.55–1.59)		1.57(1.55–1.59)	1.48(1.46–1.50)	1.34(1.31–1.38)	<0.001

Abbreviations: BMI، body mass index (calculated as weight in kilograms divided by height in meters squared); HDL-C، high-density lipoprotein cholesterol; TG, triglycerides. a: Values are age- and multivariate-adjusted means (95% confidence intervals) from generalized estimating equations to account for correlated observation. b: Adjusted for age and phaseC: Adjusted for age, phase,BMI, smoking, lipid lowering drug,education and Diabetes,Thyroid status

**Table 4:** Proportions of Participants in various Categories of dyslipidemia in baseline and 3, 6,9years after follow up.

Characteristics	Men					Women				
	Phase I (baseline)	Phase II(3y)	Phase III(6y)	Phase IV(9y)	P value trend	Phase I(baseline)	Phase II(3y)	Phase III(6y)	Phase IV(9y)	P value trend
Age adjusted analysis b										
High Cholesterol≥ 240 mg/dl	14	8.4	7.6	8.9	<0.001	20	13.3	12.6	15	<0.001
High LDL-C ≥160mg/dL	14	9	7	7	<0.001	18	12	11	10	<0.001
High TG ≥200mg/dL	29	27	25.5	25	<0.001	21	20	18	20	<0.001
High non HDL-C ≥190mg/dL	22	14	13	13	<0.001	25	15	13	12	<0.001
Low HDL-C≤40 mg/dl	67	75	62	48	<0.001	38	47	34	17	<0.001
High TG/HDL≥2.18	81	83	81	77	<0.001	64	68	64	57	<0.001
High Total Chole/HDL≥5.9	34	34	23	13	<0.001	20	21	13	7.7	<0.001
Multivariate adjusted analysis c										
High Cholel≥ 240 mg/dl	16	8	8	8	<0.001	29	15	12	10	<0.001
High LDL-C ≥160mg/dL	17	10	8	6	<0.001	25	14	11	6	<0.001
High TG ≥200mg/dL	34	29	28	23	<0.001	28	20	20	16	<0.001
High non HDL-C ≥190mg/dL	32	19	14	11	<0.001	25	15	13	12	<0.001
Low HDL-C≤40 mg/dl	67	80	72	47	<0.001	36	50	41	18	<0.001
High TG/HDL≥2.18	85	86	84	73	<0.001	70	72	66	49	<0.001
High Total Chole/HDL≥5.9	40	40	28	13	<0.001	23	22	14	9.0	<0.001

Total cholesterol had significantly higher based on TSH categorized analysis in the total population and female participants of Q3 in comparison to those of Q1, however total cholesterol in male participants was non-significant when analyzed based on TSH categories. The change in TG based on TSH categorization, was not significant in participants irrespective of sex. Participants with highest level of TSH within the normal range (Q4) had a significantly lower trend of HDL in comparison with participants of Q1, a change that was significant in total population but not separately for males and females. The change in MLDL was significantly bigger in Q3, in comparison with Q1 only for females but not in males. The trend of dyslipidemia was also adjusted based on TSH level categorization and a significant decrease in hypercholesterolemia was observed in the study population of Q3 and Q2 in comparison with Q1. Hypercholesterolemia of female participants differed statistically significant between Q3 and Q1, although male participants had no significant statistical variation between TSH quartiles. No statistically significant differences were observed for TG between TSH quartiles in male participants, female participants, or the overall population.

## Discussion

A decrescendo trend was observed in all lipid parameters of both male and female euthyroid participants, as well as those with subclinical thyroid diseases, in all residents of district 13 of Tehran during the fourth phases over 10 years. Similarly, a decrescendo trend was observed for dyslipidemia, with all lipid parameters shifting towards optimal values. There are no recent studies evaluating lipid trends with regards to thyroid function, especially in the absence of overt thyroid diseases, which signifies the importance of this study. This study was performed to examine the association between lipid trends and thyroid function. In this study we found favorable lipid trends which were persistent after adjusting for thyroid status, lipid lowering medications, age, BMI, current smoking, diabetes, hypertension, in agreement with those documented by various prospective and cross sectional studies [23–37]. Desirable downward trends of total cholesterol and LDL were also observed in France, Spain, Finland and Australia [26–30]. Initially favorable trends in total cholesterol and LDL are now reversing after 25 years of the CARDIA study without definite causes; although suggest further investigation of dietary changes and poor adherence to medications [31]. In contrast, upward trends of both total cholesterol and TG were observed in Japanese and Indian populations [32–33]; unfavorable lipid trends were completed with downward trend in HDL-C in Japanese longitudinal cohort study [34].

Less atherogenic lipid trends have been observed in different studies conducted in Europe and North America [35, 36, 37]. The adverse trend of serum lipids was also reported in Chinese school children, too [38]. Increased HDL and decreased TG levels were observed in a study by Hadaegh et al., similar to those reported in the Framingham study [27, 39–42]. Increased HDL with a flat trend in TG was observed in the CARDIA study [32].

We studied euthyroid subjects and those without overt thyroid diseases and found similar decrescendo-crescendo trends in HDL, as well as decreased TG levels. Favorable lipid trends observed in Iran, Europe and America in comparison with unfavorable lipid trend in China, India and Japan, may be linked to racial and dietary differences. Iranian diet transition from solid oils and improvement in public health information were important cofounders. Focus on people without thyroid disease, led to more distinct lipid trends in our study. High educational level was considered to be the main cause of improving lipid patterns in an Indian study [33]. Unfavorable lipid trends in China were attributed to high calorie and high fat diets [41]. Other studies have reported rising HDL without any changes in TG level [24, 26, 43]. The prevalence of hypercholesterolemia decreased by 58% in female and by 46% in male participants in our study, rates higher than those reported in previous studies that did not take thyroid function into consideration [40]. A 27% decrease in the prevalence of hypercholesterolemia was reported in an America study) 1999–2010(; Low HDL levels were reported in 12% of the female participants and 31% of the male participants of this study [44]. Our study is unique in that we adjusted lipid trends for thyroid functions and observed low HDL in 67% of the male participants and 36% of the female participants. A cross sectional study from TTS observed a statistically significant effect of normal range TSH on lipid profile, but the effect size is not clinically significant [45]. Hadaegh et al. Study of lipid trend in TLGS adults reported low HDL in 52% of the men and 26% of the women [40]. Interestingly, increases in the prevalence of obesity in parallel with those favorable lipid trends have been observed in previous studies [24, 42,43]. The prevalence of abdominal, as well as general obesity were found to have increased in the Tehranian population [46]. Mean SBP, DBP, BMI, WC, TSH level, and lipid lowering medication use has increased over time simultaneously with increasing age of participants. Diet and physical activity seem to play a significant role in lowering lipid trends. However, favorable lipid trends observed in the current study were not compatible with the findings of previous Iranian studies like the third national surveillance of risk factors of non-communicable diseases and TLGS, which observed high prevalence of low physical activity and sedentary lifestyle [47–50]. Because of missing data, physical activity was not examined in this study. Smoking is another potentially modifiable factor that affects lipid trends. Craig et al. conducted a meta-analysis to study the impact of smoking on lipid profiles and observed total hypercholesterolemia, hypertriglyceridemia, high LDL, and low HDL in smokers [51]. We observed a decrease in smoking prevalence in our participants between the 1^st^ and the 4^th^ phase of the study. An Iranian review article on smoking status (1999–2007) reported no increases in the prevalence of smoking, which may help explain favorable HDL trends observed in our study [52]. Menopause as a modulator in the association between lipid and thyroid function should be in mind for future studies [53].

Previous studies on the association of lipid parameters with thyroid function, which reported the presence of such an association had a cross-sectional design [8,10–17]; our study is the first study of trends of lipid parameters over time in relation to thyroid function. The current study did not have the limitations of previous studies (such as not checking free thyroid hormone levels) [8–17], no attention to TPOAb as a marker of thyroid autoimmunity and only one time TSH check without recheck for confirming subclinical thyroid disorders). The definition of subclinical hypothyroidism based on serum TSH levels varies widely in previous studies. Our study used a reference range of study population based on previous studies in TTS.

This study does have some limitations, including incomplete information about participants’ diets or physical activity levels. Survival bias should be considered as some participants with unfavorable lipid changes died during the study period, which could reflect favorable lipid trends in survivors. TTS was not designed to examine lipid trends in patients with subclinical thyroid diseases; therefore it was not possible to study every group of subclinical thyroid diseases. Cohort effect is inevitable. However we categorized patients based on age in order to decrease the age effect.

Strengths of our study include prospective cohort design, which evaluated a large sample of Iranians population. This is the first study of lipid trends based on thyroid function tests in the Iranian patient population.

### Conclusion

During a 10 year follow-up, decrescendo trends were observed in levels of total cholesterol, triglycerides, which were not be accounted for by the consumption of lipid lowering drugs and thyroid status. We found favorable and less atherogenic lipid trends in participants in the TTS without overt thyroid diseases. Long-term follow ups of the participants with a separate analysis of lipid trends in each thyroid disease classification is recommended for further clarification and confirmation of the current findings.

## Supporting information

S1 Table Number of lost to follow up patients. After eliminating the participants who were lost to follow up in each phase, lost data were limited as shown in the following S1 Table. Because of the small number of patients lost to follow up, estimation of selection bias and developing propensity scores were not necessary. Therefore, the selection bias is unlikely to have affected our estimations.
Abbreviations: MLDL;modified low density lipoprotein, HDL-C,high-density lipoprotein cholesterol; TG, triglycerides,TC;total cholesterol.
(DOCX)

S2 Table Age- and multivariate-adjusted mean levels a of fasting lipids by phase. Abbreviations: BMI,body mass index (calculated as weight in kilograms divided by height in meters squared); HDL-C, high-density lipoprotein cholesterol; TG، triglycerides a Values are age- and multivariate-adjusted means (95% confidence intervals) from generalized estimating equations to account for correlated observations b Model1: Adjusted for age and phase c Model2:Adjusted for age, phase, BMI, smoking, lipid lowering d*rug*, *education*, *Thyroid status and Diabetes*.
(DOCX)

S3 Table Proportions of participants in various categories of dyslipidemia in baseline and 3, 6years after follow up. Abbreviations: BMI, body mass index (calculated as weight in kilograms divided by height in meters squared); exam, examination; HDL-C, high-density lipoprotein cholesterol; TG, triglycerides a Values are age- and multivariate-adjusted means (95% confidence intervals) from generalized estimating equations to account for correlated observations
b Adjusted for age and phase.
(DOCX)
