# Correction: Trend of lipid and thyroid function tests in adults without overt thyroid diseases: A cohort from Tehran thyroid study

**DOI:** 10.1371/journal.pone.0220324

**Published:** 2019-07-23

**Authors:** Salma Ahi, Atieh Amouzegar, Safoora Gharibzadeh, Hossein Delshad, Maryam Tohidi, Fereidoun Azizi

There is an error in affiliation 3 for author Maryam Tohidi. The correct affiliation 3 is: Prevention of Metabolic Disorders Research Center, Research Institute for Endocrine Sciences, Shahid Beheshti University of Medical Sciences, Tehran, Iran.
