# Supplementary material for: Trend of lipid and thyroid function tests in adults without overt thyroid diseases: A cohort from Tehran thyroid study
Source: PLoS One. 2019 May 16;14(5):e0216389. doi: 10.1371/journal.pone.0216389 (PMC6522003; doi:10.1371/journal.pone.0216389)
Supplement: S1 Table — After eliminating the participants who were lost to follow up in each phase, lost data were limited as shown in the following S1 Table. Because of the small number of patients lost to follow up, estimation of selection bias and developing propensity scores were not necessary. Therefore, the selection bias is unlikely to have affected our estimations. Abbreviations: MLDL;modified low density lipoprotein, HDL-C,high-density lipoprotein cholesterol; TG, triglycerides,TC;total cholesterol. (DOCX) [file pone.0216389.s001.docx]

**Table S1 :( supplementary table)**

|  | Phase2 | Phase3 | Phase4 |
| --- | --- | --- | --- |
| MLDL | 9 | 3 | 0 |
| TC | 3 | 1 | 0 |
| HDL-C | 8 | 3 | 0 |
| TG | 4 | 1 | 0 |

After eliminating the participants who were lost to follow up in each phase, lost data were limited as shown in the following tableS1. Because of the small number of patients lost to follow up, estimation of selection bias and developing propensity scores were not necessary. Therefore, the selection bias is unlikely to have affected our estimations.

Abbreviations: MLDL;modified low density lipoprotein ,HDL-C,high-density lipoprotein cholesterol; TG, triglycerides,TC;total cholesterol
