# Supplementary material for: Trend of lipid and thyroid function tests in adults without overt thyroid diseases: A cohort from Tehran thyroid study
Source: PLoS One. 2019 May 16;14(5):e0216389. doi: 10.1371/journal.pone.0216389 (PMC6522003; doi:10.1371/journal.pone.0216389)
Supplement: S2 Table — Abbreviations: BMI,body mass index (calculated as weight in kilograms divided by height in meters squared); HDL-C, high-density lipoprotein cholesterol; TG، triglycerides a Values are age- and multivariate-adjusted means (95% confidence intervals) from generalized estimating equations to account for correlated observations b Model1: Adjusted for age and phase c Model2:Adjusted for age, phase, BMI, smoking, lipid lowering drug, education, Thyroid status and Diabetes. (DOCX) [file pone.0216389.s002.docx]

***TableS2. Age- and Multivariate-Adjusted Mean Levels a of Fasting Lipids by phase***

| Characteristics | Men | | | | | | | Women | | | | | | | | | |
| --- | --- | --- | --- | --- | --- | --- | --- | --- | --- | --- | --- | --- | --- | --- | --- | --- | --- |
|  | Phase I(baseline) | | Phase II(3y) | Phase III(6y) | | P value trend | | Phase I(baseline) | Phase II(3y) | | | Phase III(9y) | | | P value trend | | |
| Age adjusted analysis b | | | | | | | | | | | | | | | | | |
| Total cholesterol, mg/dL | 197.89(196.04-199.74) | 187.40(185.58-189.22) | | | 186.93(185.08-188.78) | | <0.001 | 209.98(208.24-211.72) | | 193.85(192.14-195.56) | | | 190.51(188.77-192.25) | | | <0.001 | |
| HDL-C, mg/dL | 37.39(36.95-37.83) | 35.72(35.29-36.15) | | | 38.83(38.39-39.26) | | <0.001 | 44.36(43.88-44.84) | | 42.13(41.66-42.60) | | | 46.23(45.75-46.71) | | | <0.001 | |
| TG, mg/dL | 5.01(4.98-5.04) | 4.97(4.95-5.00) | | | 4.96(4.93-4.98) | | <0.001 | 4.89(4.87-4.92) | | 4.82(4.80-4.84) | | | 4.77(4.75-4.80) | | | <0.001 | |
| LDL M,mg/dL | 126.89(125.39-128.39) | 119.68(118.20-121.15) | | | 116.81(115.32-118.31) | | <0.001 | 133.35(131.93-134.77) | | 121.87(120.48-123.26) | | | 115.98(114.56-117.4) | | | <0.001 | |
| TG/HDL-C | 1.41(1.38-1.45) | 1.43(1.39-1.46) | | | 1.32(1.29-1.36) | | <0.001 | 1.13(1.10-1.16) | | 1.11(1.10-1.14) | | | 0.97(1.08-1.14) | | | <0.001 | |
| Tc/HDL | 1.67(1.65-1.68) | 1.66(1.65-1.67) | | | 1.57(1.56-1.59) | | <0.001 | 1.55(1.54-1.57) | | 1.53(1.51-1.54) | | | 1.42(1.41-1.43) | | | <0.001 | |
| Multivariate adjusted | | | | | | | | | | | | | | | | | |
| Total cholesterol, mg/dL | 203.72(201.63-205.81) | 187.09(185.26-188.92) | | | 186.18(184.31-188.05) | | <0.001 | 212.58(210.59-214.58) | | | 192.36(190.60-194.11) | | | 188.72(186.92-190.52) | | | <0.001 |
| HDL-C, mg/dL | 38.22(37.72-38.72) | 35.58(35.15-36.00) | | | 38.85(38.42-39.28) | | <0.001 | 45.78(45.20-46.35) | | | 42.17(41.67-42.67) | | | 46.28(45.7-46.80) | | | <0.001 |
| TG, mg/dL | 5.06(5.03-5.09) | 4.98(4.95-5.00) | | | 4.96(4.92-4.97) | | <0.001 | 4.88(4.85-4.90) | | | 4.80(4.77-4.83) | | | 4.75(4.73-4.77) | | | <0.001 |
| Modified LDL,mg/dl | 130.49(128.79-132.19) | 119.35(117.87-120.84) | | | 116.31(114.79-117.83) | | <0.001 | 134.57(132.95-136.19) | | | 120.89(119.45-122.32) | | | 114.62(113.14-116.09 | | | <0.001 |
| TC/HDL-C | 1.43(1.40-1.47) | 1.43(1.40-1.46) | | | 1.31(1.27-1.34) | | <0.001 | 1.00 (1.05-1. 11) | | | 1.00(1.06-1.11) | | | 0.95(0.92-0.97) | | | <0.001 |
| TC/HDL | 1.67(1.66-1.69) | 1.66(1.65-1.68) | | | 1.57(1.55-1.58) | | <0.001 | 1.54(1.52-1.55) | | | 1.52(1.51-1.53) | | | 1.41(1.0-1.42) | | | <0.001 |

Abbreviations: BMI,body mass index (calculated as weight in kilograms divided by height in meters squared); HDL-C, high-density lipoprotein cholesterol; TG، triglycerides a Values are age- and multivariate-adjusted means (95% confidence intervals) from generalized estimating equations to account for correlated observations b Model1: Adjusted for age and phase c Model2:Adjusted for age ,phase, BMI, smoking, lipid lowering d*rug, education, Thyroid status and Diabetes*
