# Supplementary material for: Trend of lipid and thyroid function tests in adults without overt thyroid diseases: A cohort from Tehran thyroid study
Source: PLoS One. 2019 May 16;14(5):e0216389. doi: 10.1371/journal.pone.0216389 (PMC6522003; doi:10.1371/journal.pone.0216389)
Supplement: S3 Table — Abbreviations: BMI, body mass index (calculated as weight in kilograms divided by height in meters squared); exam, examination; HDL-C, high-density lipoprotein cholesterol; TG, triglycerides a Values are age- and multivariate-adjusted means (95% confidence intervals) from generalized estimating equations to account for correlated observations b Adjusted for age and phase. (DOCX) [file pone.0216389.s003.docx]

***Table S3. Proportions of Participants in various Categories of dyslipidemia in baseline and 3, 6years after follow up***

| Characteristics | Men | | | | Women | | | |
| --- | --- | --- | --- | --- | --- | --- | --- | --- |
|  | Phase I(baseline) | Phase II(3y) | Phase III(9y) | P value trend | Phase I(baseline) | Phase II(3y) | Phase III(9y) | P value trend |
| Age adjusted analysis b | | | | | | | | |
| High Cholesterol ≥ 240 mg/dl | 13 | 8 | 7 | <0.001 | 24 | 13 | 10 | <0.001 |
| High M LDL-C ≥160mg/dL | 14 | 8 | 7 | <0.001 | 21 | 12 | 9 | <0.001 |
| High TG ≥200mg/dL | 28 | 27 | 25 | <0.001 | 24 | 19 | 16 | <0.001 |
| High non HDL-C ≥190mg/dL | 25 | 17 | 12 | <0.001 | 20 | 13 | 12 | <0.001 |
| Low HDL-C≤40 mg/dl | 67 | 75 | 62 | <0.001 | 40 | 46 | 34 | <0.001 |
| High TG/HDL≥2.18 | 82 | 83 | 80 | <0.001 | 64 | 68 | 64 | <0.001 |
| High Total Chole/HDL≥5.9 | 34 | 34 | 24 | <0.001 | 22 | 20 | 11 | <0.001 |
| Multivariate adjusted analysis c | | | | | | | | |
| High Cholesterol≥ 240 mg/dl | 15 | 8 | 7 | <0.001 | 25 | 12 | 10 | <0.001 |
| High M LDL-C ≥160mg/dL | 15 | 8 | 7 | <0.001 | 21 | 12 | 8 | <0.001 |
| High TG ≥200mg/dL | 31 | 27 | 24 | <0.001 | 24 | 18 | 16 | <0.001 |
| High non HDL-C ≥190mg/dL | 28 | 16 | 12 | <0.001 | 23 | 13 | 12 | <0.001 |
| Low HDL-C≤40 mg/dl | 64 | 76 | 63 | <0.001 | 35 | 46 | 33 | <0.001 |
| High TG/HDL ≥2.18 | 65 | 76 | 63 | <0.001 | 68 | 67 | 60 | <0.001 |
| High Total Cholesterol /HDL ≥5.9 | 36 | 34 | 22 | <0.001 | 21 | 19 | 11 | <0.001 |

Abbreviations: BMI, body mass index (calculated as weight in kilograms divided by height in meters squared); exam, examination; HDL-C, high-density lipoprotein cholesterol; TG, triglycerides a Values are age- and multivariate-adjusted means (95% confidence intervals) from generalized estimating equations to account for correlated observations

b Adjusted for age and phase
